# Mechanisms of Action of a Web-Based Intervention With Health Professional Support to Increase Adherence to Nebulizer Treatments in Adults With Cystic Fibrosis: Qualitative Interview Study

**DOI:** 10.2196/16782

**Published:** 2020-10-06

**Authors:** Sarah J Drabble, Alicia O'Cathain, Alexander J Scott, Madelynne A Arden, Samuel Keating, Marlene Hutchings, Chin Maguire, Martin Wildman

**Affiliations:** 1 School of Health and Related Research University of Sheffield Sheffield United Kingdom; 2 Centre for Behavioural Science and Applied Psychology Sheffield Hallam University Sheffield United Kingdom; 3 Sheffield Adult CF Centre Sheffield Teaching Hospitals Sheffield United Kingdom

**Keywords:** United Kingdom, cystic fibrosis, adherence, compliance, health behavior, psychological theory, process evaluation, qualitative research, interview, patient portals, telehealth

## Abstract

**Background:**

Adherence to nebulizer treatments in adults with cystic fibrosis (CF) is often low. A new complex intervention to help adults with CF increase their adherence to nebulizer treatments was tested in a pilot randomized controlled trial (RCT) in 2 UK CF centers. Patients used a nebulizer with electronic monitoring capabilities that transferred data automatically to a digital platform (CFHealthHub) to monitor adherence over time and to a tailored website to display graphs of adherence data and educational and problem-solving information about adherence. A trained interventionist helped patients identify ways to increase their adherence.

**Objective:**

This study aims to explore the mechanisms of action underpinning the intervention.

**Methods:**

A qualitative interview study was conducted concurrently with a pilot RCT. In total, 25 semistructured interviews were conducted with 3 interventionists at 2 time points, 14 patients in the intervention arm of the trial, and 5 members of the multidisciplinary teams offering wider care to patients. A framework approach was used for the analysis.

**Results:**

The intervention was informed by a theoretical framework of behavior change. There was evidence of the expected behavior change mechanisms of action. There was also evidence of additional mechanisms of action associated with effective telehealth interventions for self-management support: relationships, visibility, and fit. Patients described how building a relationship with the interventionist through face-to-face visits with someone who cared about them and their progress helped them to consider ways of increasing adherence to medication. Rather than seeing the visibility of adherence data to clinicians as problematic, patients found this motivating, particularly if they received praise about progress made. The intervention was tailored to individuals, but there were challenges in how the intervention fitted into some patients’ busy lives when delivered through a desktop computer.

**Conclusions:**

The mechanisms of action associated with effective telehealth interventions for self-management operated within this new intervention. The intervention was modified to strengthen mechanisms of action based on these findings, for example, delivery through an app accessed via mobile phones and then tested in an RCT in 19 UK CF centers.

**Trial Registration:**

International Standard Randomized Controlled Trial Number 13076797; http://www.isrctn.com/ISRCTN13076797

## Introduction

### Background

Adherence to medication for chronic conditions is a complex issue that is sometimes addressed by digital, web-based, mobile, and telehealth interventions [1-3]. We developed a new intervention involving a web-based adherence monitoring system with health professional support to help adults with cystic fibrosis (CF) increase their adherence to nebulizer treatments. Although not promoted as telehealth, the intervention has key components of web-based monitoring and web-based behavior change materials for use with the chronic condition of CF. CF is a genetic life-limiting condition affecting approximately 100,000 people worldwide [4] in which mucus builds up in the lungs, digestive system, and other organs, leading to difficulty in breathing, respiratory infections, and ultimately death. Patients with CF need to take preventative treatments such as antibiotics and mucolytics through nebulizers and airway clearance, often alongside many other treatments creating a complex, onerous treatment regime [5,6]. Adherence to nebulizer treatments is often low, with objectively measured adherence found to average 36% compared with 80% estimated by patients [7]. Low adherence to nebulizer treatments has been linked to worse health outcomes, including decreased lung function, increased pulmonary exacerbations requiring treatment with intravenous antibiotics, and higher service costs [8-11].

We tested the intervention in a feasibility study that included a pilot randomized controlled trial (RCT) and a mixed methods process evaluation. We found that a full-scale RCT was feasible with numbers of pulmonary exacerbations as the primary outcome. The pilot RCT identified that mean changes to adherence, a key secondary outcome, were 10% higher in the intervention arm (95% CI −5.2 to 25.2) [12]. The mixed methods process evaluation found that the intervention was feasible and acceptable to patients, interventionists, and health care professionals in the multidisciplinary teams (MDTs) offering wider care to patients with CF. As part of the process evaluation, we conducted qualitative interviews with patients and interventionists. One of the aims of this qualitative research was to consider the proposed mechanisms of action underpinning the intervention in practice. The mechanisms of action explain how the intervention components might work to produce an outcome [13,14] and how any theoretical model might be working within the intervention. Qualitative research can be undertaken during the development and feasibility phases of developing complex interventions [14,15] to help researchers understand how a complex intervention with multiple and interacting components might work [16]. Qualitative research within process evaluations can help researchers understand mechanisms of action by looking for unanticipated and complex causal pathways through the intervention or unanticipated consequences of the intervention [17,18], which is useful in refining the intervention before a full-scale evaluation.

### Description of the Intervention

**Figure 1:**
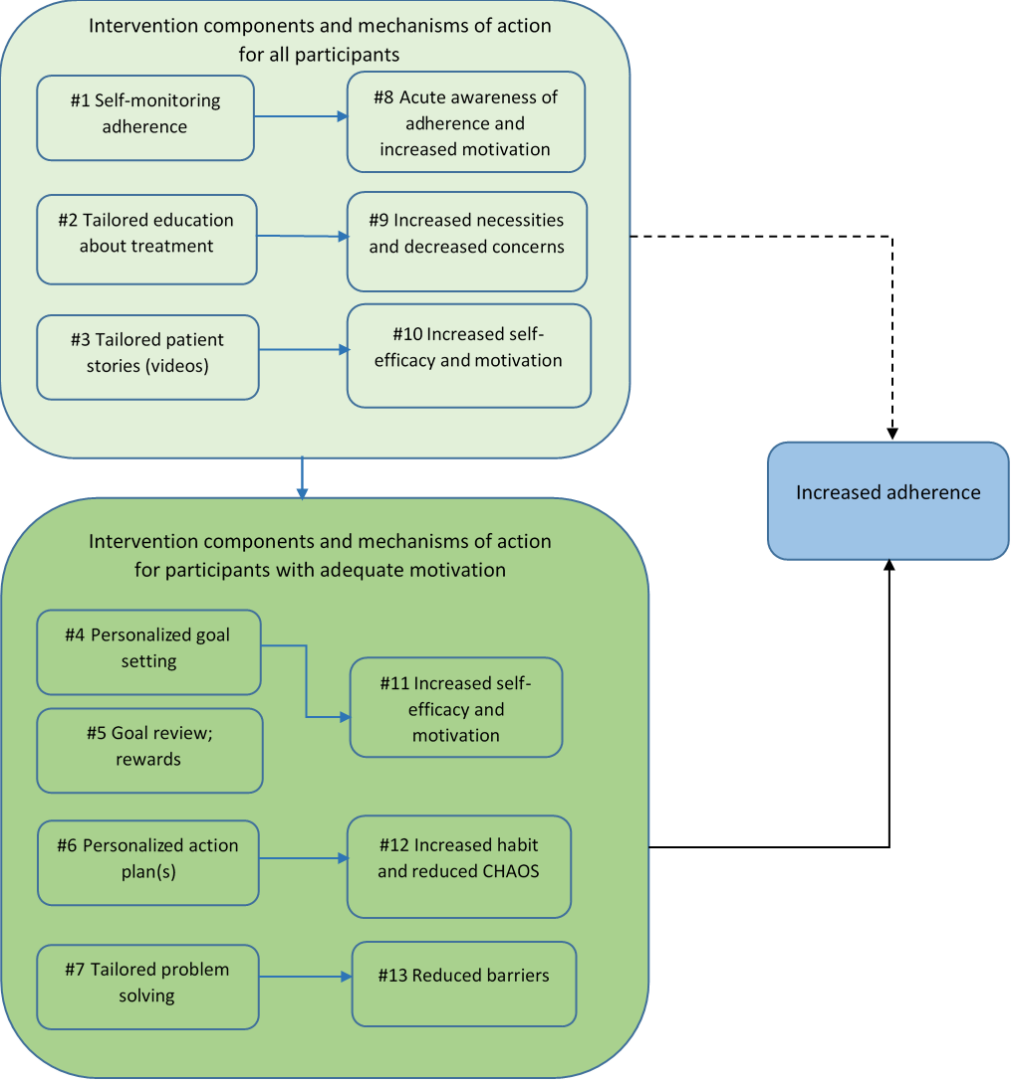
Theoretical model of behavior change to increase adherence in adults with cystic fibrosis. The Confusion, Hubbub, and Order Scale is a 6-item measure of life chaos. CHAOS: Confusion, Hubbub, and Order Scale.

The intervention is complex, with a number of interacting components [16]. A study by Arden et al (unpublished data, 2020) provides more details on the intervention and development processes. In summary, an eTrack nebulizer (PARI, Pharma GmbH) with electronic monitoring capabilities sends timestamped inhalation data to a 2net Hub (Qualcomm). This enables real-time monitoring of adherence via securely stored data on CFHealthHub servers. Adherence data are displayed on the CFHealthHub website and are visible to patients and health professionals delivering the intervention. The intervention also comprises a manualized behavior change component that is designed to help patients increase their adherence. This is underpinned by a theoretical model of behavior change (Figure 1) based on the idea that reflective motivation, in which people rationally weigh up the perceived necessity of a behavior against the perceived concerns they have about treatment, underpins adherence behavior. Therefore, some participants would need to address motivation before other strategies, such as action planning, could be successfully used. The model of behavior change is part of a larger logic model [12], informed by qualitative interviews with adults with CF about their adherence behavior [19,20] mapped to the Theoretical Domains Framework [21] and behavior change wheel [22] and developed using a person-based approach [23]. The behavior change intervention is delivered by trained health professionals (interventionists) in a series of face-to-face and telephone meetings with patients. Interventionists help patients to increase their adherence to nebulizer treatments by choosing components of the behavior change intervention to address an individual’s unique issues identified through baseline questionnaires and discussions during intervention visits and placed into a toolkit area of the CFHealthHub website. The interventionists in the pilot RCT were clinicians employed to deliver the intervention. The number and location of intervention visits are tailored to patients’ needs.

Figure 1 [24] shows how the model splits the behavior change components into components for all participants (including those with low motivation) and for those with adequate motivation (#1 to #7) and how those components link to mechanisms of action (#8 to #13) that lead to increased adherence. Table 1 describes the different components and the proposed mechanisms of action in more detail and how they are delivered within the intervention. The numbers in brackets in the table are linked to the numbers in Figure 1.

**Table 1:** Components of the intervention and proposed mechanisms of action.

Components		Description of component and *proposed mechanisms of action*^a^	Delivery
**For all participants**			
	Self-monitoring adherence (#1)	Patients self-monitor their adherence by reviewing graphs of adherence data by month, week, or time of the day in CFHealthHub. A simple traffic light system quickly indicates if patients have hit their agreed target for treatments (green), have done some treatments but not hit their target (amber), or have completed no treatments (red). *This enables patients to understand their adherence patterns leading to awareness of adherence and increased motivation to adhere* (#8)	Graphs showing red, amber, and green charts of adherence data on CFHealthHub and tables showing the number of treatments completed compared with prescription data
	Tailored education about treatment (#2)	Interventionist identifies issues with understanding treatments and unhelpful beliefs about different aspects of treatment. *Patients can see the need to undergo treatment (increased necessities) and reduce any concerns they have about treatments* (#9)	Baseline questionnaires and discussions with patients used by interventionists to identify issues before visits Education section on CFHealthHub with information about CF^b^ and videos about how treatments work and links to other websites. Interventionists use questionnaires and discussions to identify where patients lack knowledge or have misconceptions
	Tailored patient stories (videos; #3)	Other patients with CF talking about how they overcame different barriers to give patients information from peers or someone like them. *This allows patients to see how they can find solutions to problems (increased self-efficacy) and that it is possible to change (increased motivation*; #10)	Section of CFHealthHub includes *talking heads* videos that address adherence barriers. Interventionists use these with patients if they address an issue that patients experience
**For patients with sufficient motivation**			
	Personalized goal setting (#4)	Interventionist works with patient to set goals for adherence against prescription and life goals that may help adherence behavior. *This allows patients to work toward a target (increased self-efficacy) and see how adherence is linked to their life goals (increased motivation to adhere*; #11)	Graphs in CFHealthHub measure actual adherence against personalized adherence goals and can be tracked by interventionists Interventionists work with patients during visits to set personalized adherence and life goals if appropriate
	Goal review, rewards (#5)	A review of progress toward goals with the patient helps to increase self-efficacy. *Optional notifications of achievements against progress help patients to increase motivation to adhere* (#11)	Goals are reviewed during discussions between patients and interventionists and barriers are identified Patients receive messages on mobile phones or in CFHealthHub to provide motivation to keep going with behavior changes
	Personalized action plans (#6)	Patients create personalized action plans around daily routines to build adherence *that increases habits and reduces chaos* (#12)	Action plans are made by patients with help from interventionists. Action plans are written in a section in CFHealthHub for patients to refer to
	Tailored problem solving (#7)	Solutions to common problems with adherence, for example, what to do when you go on holiday. *These allow patients to solve problems, reducing barriers to adherence* (#13)	Questionnaires and discussions between interventionists and patients identify practical barriers that make adherence difficult. These are stored on a problem-solving section on CFHealthHub

^a^Proposed mechanisms of action are shown in italics.

^b^CF: cystic fibrosis.

Whilst considering the proposed mechanisms of action in Table 1 in a qualitative study, we identified 3 mechanisms of action (relationships, visibility, and fit) that Vassilev et al [25] found in a realist review of effective telehealth interventions that explain how telehealth supports self-management of long-term conditions. First, effective telehealth interventions should consider how *relationships* with health professionals and peers are enabled or inhibited. Second, the intervention should enable the *visibility* of symptoms to the self and others and provide feedback to increase knowledge, motivation, and empower patients, thus enabling reinforcement of behavior change. Finally, successful interventions should *fit*, or at least not disrupt, the everyday lives and routines of patients, including their existing skills and knowledge. The study by Vassilev et al [25] is transferable to this intervention because there are key components of telehealth, namely, the intervention has web-based monitoring and web-based behavior change materials, the intervention is concerned with self-management (adherence to nebulizer treatments), and the condition being studied, CF, is a long-term condition. Although relationships, fit, and visibility are implicit in the stated intervention components, the ways in which they might work in the intervention as potential mechanisms of action were not fully articulated; thus, their importance to the potential effectiveness of the intervention needed further exploration. In this study, we explore the mechanisms of action operating in the intervention by confirming how the expected mechanisms of action identified in Table 1 worked in the intervention and by showing how important the mechanisms of action (relationships, visibility, and fit) identified in the review by Vassilev et al [25] of telehealth for self-management support were to facilitate the potential effectiveness of this intervention.

## Methods

### Study Design

We undertook a feasibility study in preparation for a full-sized RCT. The study consisted of a mixed methods process evaluation undertaken concurrently with a pilot RCT in 2 UK CF centers. In total, 33 adults with CF received the intervention for 5 months [12]. The process evaluation addressed the feasibility and acceptability of the intervention and the RCT and the mechanisms of action of the intervention. The qualitative component of the process evaluation allowed exploration of the mechanisms of action at play, and this is the focus of this paper. The study received ethical approval from the London Brent Research Ethics Committee (16/LO/0356).

### Participants

We conducted 25 semistructured face-to-face interviews, with patients in the intervention arm of the RCT (n=14), interventionists (n=3 at 2 time points), and members of the wider MDT offering care to patients with CF (n=5). We interviewed more patients in RCT site 1 (n=8) than in RCT site 2 (n=6), because fewer patients agreed to be interviewed at site 2. Sociodemographic data for patient participants are shown in Table 2.

**Table 2:** Sociodemographic information for patient participants (n=14).

Characteristics		Participants, n (%)
**Gender**		
	Male	9 (64)
	Female	5 (36)
**Age (years)**		
	≤18	1 (7)
	19-25	6 (43)
	31-40	3 (21)
	>40	4 (29)
**Deprivation (IMD^a,b^ quintile)^c^**		
	1	6 (43)
	2	1 (7)
	3	2 (14)
	4	3 (21)
	Missing data	2 (14)
**Baseline objective adherence^c,d^**		
	High	3 (21)
	Moderate	1 (7)
	Low	2 (14)
	Very low	5 (36)
	Missing data	3 (21)

^a^IMD: Index of Multiple Deprivation by Postcode [26].

^b^The Index measures relative deprivation by UK postal code stratifying into 5 quintiles, where 1 is the most affluent and 5 is the most deprived. By entering a postal code, it is possible to obtain an indication of the deprivation level where an individual lives.

^c^The percentage values for deprivation and baseline objective adherence add up to 99% due to rounding issues.

^d^Mean unadjusted objective adherence over the previous 6 months measured from chipped i-neb nebulizers: high (≥80.0%), moderate (50.1%-79.9%), low (25.1%-50.0%), and very low (≤25.0%) [11,27].

We interviewed all 3 interventionists. They had different backgrounds: 2 had worked as physiotherapists within the multidisciplinary health care team who provided care to patients with CF in that center and one was a psychologist who had not previously worked in a CF setting. The MDT members were from a mix of disciplines, including respiratory consultants and physiotherapists.

### Recruitment

During consent for the pilot RCT, 28 of the 33 intervention patients consented to be approached for an interview. We invited participants by letter or email and followed up by telephone or email. Of the 28 available patients, 16 consented to interview, 2 declined, 1 withdrew from the full study, 1 died during the study, we were unable to contact 7 patients, and we did not approach 1 patient because of time constraints. Of the 16 consented, 14 were interviewed and 2 patients canceled their interviews. The 3 interventionists consented to be interviewed twice during the study, toward the beginning and the end of the pilot RCT. MDT participants were recruited through the interventionists at each site. We obtained informed written consent from all participants before the interview.

### Data Collection

Interviews were conducted by SD, AS, and SK. Patients were interviewed face-to-face in their homes or the hospital depending on preference, whereas interventionists and MDT interviews were conducted at the hospital, except one that was by telephone. We used topic guides as the basis for the interviews, with questions about the acceptability of different aspects of the intervention and RCT, and explored which aspects of the intervention participants perceived to be important in increasing adherence to nebulizer treatments (mechanisms of action). Interviews were also open enough to allow patients to describe their approaches to adherence and any challenges they faced. The interviews lasted between 11 and 102 min, averaging 56 min. Interviews were digitally audio-recorded and transcribed verbatim. We removed identifying data before analysis and gave participants an identification number to maintain anonymity.

### Data Analysis

SD and AS conducted the initial coding. We initially derived a coding framework [28] based on the Theoretical Domains Framework categories related to adherence [19] and the key aspects of process evaluation (context, mechanisms, and implementation) [17]. After initial familiarization with the data, SD added the telehealth mechanisms of action by Vassilev to the coding framework, because they were evident in the data [25]. SD and AS read the transcripts and coded them against the framework using NVivo software, allowing for other themes to be identified in the data set that did not fit the framework. After the initial coding, SD worked with MA to discuss the relationship between the telehealth mechanisms by Vassilev (relationships, visibility, and fit) and the expected mechanisms of action based on behavior change theory. Trustworthiness was addressed through: researchers writing reflections after each interview, discussion of the interviews between SD, AS, and SK during data collection, discussion of the findings between SD and AC to develop the analysis, and presentations to the wider research team. We present quotes from patients in the intervention (P), interventionists (INT), and members of the MDT to provide examples of the points we are making.

## Results

In the qualitative interviews, we found evidence of the expected behavior change mechanisms of action. The mechanisms of action that appeared to work during the pilot RCT are presented in Table 3.

In a study of the mechanisms of action in telehealth interventions for self-management support, Vassilev et al [25] emphasized the importance of some mechanisms of action (relationships, visibility, and fit). These mechanisms are implicit in some of the behavior change mechanisms of action discussed earlier, but Vassilev’s work identified how important they were in facilitating the potential effectiveness of this intervention. These are discussed below.

**Table 3:** Description of how behavior change components and mechanisms of action worked in the intervention.

Component and mechanism of action	Evidence	Examples of quote
**Self-monitoring (#1) leading to acute awareness of adherence and increased motivation to adhere (#8)**		
	Patients found it motivating to see the green bars on their graphs because it was a quick and easy way to see their progress	“I’m very much more conscientious of how much I’m doing it and almost kind of realized the importance of doing it a lot more.” (P1, site 2)
	Some patients reported self-monitoring leading to increased awareness of adherence level although this did not always lead to increased motivation to adhere to treatment or change to adherence behavior	“They kind of say in theory it’s a good idea and they like it but somehow they’re not doing it.” (INT2, site 2)
**Tailored education about treatment (#2) leading to increased necessities and decreased concerns about doing treatments (#9)**		
	Educational components such as the treatment videos were perceived by clinicians and patients as beneficial and a trusted information source	“People have said that its nice having something that you know has been prepared by […] professionals so you know the information is accurate without it being scary.” (INT1, site 2)
	Some patients found it difficult to translate the education into action. The pages were not accessed frequently outside meetings with interventionists	“I don’t know what it is but if someone tells me to do something, yes I take it on board but I’m not very good at putting that into action.” (P2, site 1)
**Tailored patient stories (videos; #3) leading to increased self-efficacy and motivation to adhere (#10)**		
	The *talking heads* videos did not appear to increase self-efficacy and motivation (#30) for most patients in the sample because they feared comparison with others with CF	“I don’t need to listen to somebody feeling sorry for themselves.” (P5, site 1)
	Interventionists sometimes did not share the videos because they found it difficult to know the content of each video in detail and were concerned about sharing videos that could upset patients	“Some of them said about the videos of people ‘I've not looked because I don't want to see and compare myself to that person’” (INT1, site 1)
**Personalized goal setting (#4) leading to increased self-efficacy and motivation to adhere (#11)**		
	Some participants found it motivating to set targets that they could work to achieve	“Again, the graphs and stats and things like that […] motivate you, keep you in the right place.” (P4, site 2)
	Some patients preferred to set lower, achievable goals	“They know they are clearly struggling to do what's required so some people were quite happy to be told they could do less [than 100%].” (INT2, site 2)
	Some low adherers wanted to set unrealistically high goals that were unachievable	“The one I did this morning, we put her at 100% and I said that’s quite high and she was like, no, her words were like, all or nothing.” (INT1, site 2)
	Some high adherers did not set goals	“I do manage [treatments] practically twice a day so [the interventionist] didn’t really set me any goals.” (P1, site 2)
**Goal review, rewards (#5) leading to increased self-efficacy and motivation to adhere (#11)**		
	Notifications were unavailable during the pilot, but some patients thought they would help them adhere	“I want to receive reminders—target achieved.” (P3, site 2)
**Personalized action plans (#6) leading to increased habits and reduced chaos (#12)**		
	Interventionists and some patients described how action plans were beneficial for a minority of patients to create a habit or routine, but some patients disliked them because they did not want to form habits	“It [has] helped because now I’m starting to think of things that I can link with […] it’s making me make a conscious effort toward helping my health.” (P2, site 2) “My life is up and down you know and saying for every 5 days a week for example I’m going to do this at this time it don’t work for me at all.” (P2, site 1)
	Patients and interventionists perceived that the action plans could sometimes feel simplistic	“[A patient] said to be honest I did feel like you know, I’m not a child, that was her reaction but she was quite nice about it” (INT 2, site 2)
**Tailored problem solving (#7) leading to reduced barriers to adherence (#13)**		
	Most patients interviewed did not access the problem-solving part of the website outside the meetings with interventionists but found the resources useful if they did encounter a problem	“If you’re getting in a mess with the equipment, bits and bobs like that—the few things that I investigated on that were really quite helpful.” (P4, site 2)

### Relationships: Building a Relationship With the Interventionist

A trained interventionist delivered the intervention through a series of tailored meetings where patients and interventionists discussed all aspects of adherence behavior. The way the interventionists communicated was intended to encourage open and honest communication about adherence, from which a realistic understanding of the barriers that patients faced in their daily lives could emerge. The importance of the style of communication was addressed in the intervention manual and during the interventionists’ training program. In the pilot RCT, patients and interventionists emphasized the importance of these interactions between interventionists and patients in helping them to improve their adherence. In particular, they valued where the interactions occurred and how interventionists communicated with patients.

Meetings in a patient’s home were face-to-face, lasting up to an hour. These meetings were highly valued by some patients because patients perceived that the time given and effort made to travel to a patient’s home by the interventionist were indications of their interest in the patient’s adherence:

I feel like there’s a lot more care in that they’re more interested in the patient.

P8, site 1

The fact that visits were home-based was also important to some patients because this venue provided a safer space in which patients had time to talk in-depth about adherence. Interventionists also appreciated being able to find out about the lives of patients and some of the barriers they faced by seeing their living conditions. For some patients, this was an important first step in reflecting on their adherence behavior and trying to understand and identify potential barriers. It also demonstrated to patients the importance of focusing on their adherence:

Physically coming to your home to talk to you about it, makes you think right, okay [...] I’m focused here.”

P3, site 1

Face-to-face meetings also gave the opportunity for informal talk, which helped to establish a rapport between patients and interventionists. One interventionist felt that the script-like nature of the intervention could prevent relationship building, so they tried to make the intervention visits flow by including *general chit-chat* and letting patients make conversation at their own pace. This contributed to patients feeling like the interventionists displayed a caring attitude toward them:

She’s really nice and I think she’s very caring, for me to see her face-to-face has helped.

P8, site 1

This caring attitude was an important aspect in building a relationship that had the potential to help the patients increase their adherence by allowing them to be open about the barriers they faced to adherence.

### Visibility: Self and Others Monitoring Adherence

Visibility was a key aspect of the intervention. Self-monitoring of real-time adherence data in the form of graphs and tables against personalized goals, in combination with open discussions about adherence behavior (see *Relationships: Building a Relationship With the Interventionist*), was intended to raise the visibility of adherence for patients and clinicians by aiding memory about specific instances of adherence or nonadherence. Discussions could then focus on identifying barriers to adherence, which could be addressed to improve self-efficacy. On the other hand, although monitoring of data by the multidisciplinary health care team was part of the intervention, it was not planned as a central component of the intervention and was not linked to a mechanism of action to increase adherence directly. In the pilot RCT, it was apparent that visibility to the self was an important motivator to improve adherence while visibility to others, that is, people they felt accountable to, could also be a motivator.

In the pilot RCT, we found that visibility to the self-operated as expected through self-monitoring (Table 3). Visibility to others operated when the intervention allowed the interventionist, and with patient consent, their multidisciplinary health care team, to see adherence data. Although most patients we interviewed chose to share their data with their health care team, some patients did not always like being monitored by others. Indeed, some members of the multidisciplinary health care team worried that the intervention could be perceived as unwelcome, described as *Big-Brotherish* by one interviewee (MDT1, site 2), involving the health care team checking up on patients or telling them off.

Patients who chose to share their data described how others monitoring adherence could make them accountable to someone else in their health care team. They also described how others’ monitoring motivated them to adhere through a desire to look good in front of others:

If I go to click and I know that I haven’t done it, I know that my doctor knows that I haven’t done it. So I suppose in a weird way that’s kind of how I’m motivated.

P4, site 1

Offering praise to patients to increase their adherence could further motivate patients. For example, although this was not specified as part of the intervention, one of the interventionists would text patients with praise or encouragement to keep going if graphs showed improvements:

I just periodically look at the tables to see how they’re doing and then I text them a response of what I’ve seen and that’s been quite nice you know, they’ve sort of liked that, I’ll say “oh I’ve just checked and you’re doing really well” you know “keep it up” sort of thing.

INT1, site 2)

Patient 3 (site 2): *I like knowing that I’m being, not monitored and stuff, but because of when that text from [the interventionist] she was like “you’re doing absolutely amazing,” and because it is hard and […] bringing in the reality of illness every day and then saying “oh you’re doing amazing.”*

Interviewer*: Mm so that extra support as well that someone is keeping an eye out*

Patient 3: *Yeah, that you’re not alone.*

### Fit: A Better Fit for Others?

The intervention was developed to be tailored to individual patients so that the education about treatments, setting goals and action plans, and problem solving fitted patients’ specific needs. Particular content identified by the interventionists could be added to the toolkit area of the website, offering a tailored space for each patient. In addition, the intervention was tailored to patient circumstances by offering options for interventionist visits in the patient’s home, hospital, or by telephone.

In the pilot RCT, patients valued this tailoring and believed that it facilitated adherence improvement because not every patient had the same experience or background, so it was important that the intervention allowed patients to focus on the aspects that were most useful to them:

I think there are different age groups, there’s different educational backgrounds, it’s different people with different levels of intelligence. So you have to kind of throw it in there that covers all those people and then they opt in and out of what suits them best.

P3, site 1

Although patients appreciated the ability to tailor the intervention, it was important to them that this tailoring was patient-led, that is, tailored to the patient’s needs rather than the interventionist suggesting or even dictating what targets and goals should be set by the patient:

Sometimes going with what the patient feels, y’know if you think it might be a good idea for them to have an action plan or a coping plan and some of them just don’t really want to do that, even though you think it might be valuable for them.

INT1, site 2

One patient with high baseline adherence appreciated how the interventionist recognized that they did not need to set a goal to improve adherence, that is, they did not believe they needed some aspects of the intervention:

She said there wasn’t really a point because I was already so good at doing them.

P1, site 2

However, there were problems around fit with patients’ perceived needs and lives, with some of the patients seeing the intervention as a better fit for others rather than themselves. Although most patients in the sample believed the intervention was useful, some patients with baseline high adherence did not see the need for setting action plans, but believed that those struggling with adherence would benefit from these. Some patients also perceived that education, problem solving, and video components were more useful for other people, such as younger or newly diagnosed patients with less knowledge:

Well it would be for, I think for younger people but obviously I know what CF is, I know the ins and outs of it.

P1, site 2

Importantly, some patients believed that the intervention did not fit their lifestyles because they were busy and could not access the intervention away from their desktop. For example, although the toolkit area was appreciated by patients, interventionists noticed that they were unlikely to look at the materials outside the meetings. Patients described not having time to log into the website and preferring an app to access the website flexibly when away from home. There was also an issue around fit with priorities in life. For one patient, adherence was low down their list of priorities compared with work, although there were signs that the intervention had started to help them challenge that belief:

Work is a helluva lot more important and I don’t see my health as important as it should be, so my treatments are probably quite low on my ranking of things [...], but obviously I need to start changing that because it’s not turning out well.

P2, site 2

There was a view among health professionals that the intervention fitted the needs and priorities of patients with moderate baseline adherence rates because high adherers were adhering without the intervention, and low adherers, who perhaps had the most need, were difficult to engage:

The girl who is the worst adherer out of all those who’s got the chaotic life was the one who thought there was absolutely no need to do any plans. But I think it’s almost like her lifestyle is that chaotic that its quite difficult to make a plan. She really didn’t see the need which I thought was a shame because I thought it might have been helpful to her.’

INT2, site 2

## Discussion

### Principal Findings

In this study, we described how components and mechanisms of action for behavior change planned within a new intervention potentially helped patients to increase adherence to nebulizer treatments. We also described how mechanisms of action for effective telehealth interventions for self-management support (relationships, visibility, and fit) [25] were operationalized and valued by patients using the intervention. Patients described how building a relationship with the interventionist through face-to-face visits in the home with someone who cared about them and their progress helped them to consider ways of increasing adherence to medication. Rather than seeing the visibility of adherence data to clinicians as problematic, patients found this motivating because it made them accountable to someone else, particularly if they received praise about progress made. Although the intervention was tailored to individuals, there were challenges in how the intervention fitted into some patients’ busy lives, particularly when delivered through a desktop computer. The intervention was perceived as a better fit for other patients, for example, those who were younger or less knowledgeable. Interventionists identified a middle group of patients with moderate baseline adherence who might benefit more from the intervention.

### Comparison With the Literature

Sitting down face-to-face with an interventionist provided participants with someone who they felt cared about them, providing motivation to increase their adherence. This echoes previous research that has shown how the human aspect of a telehealth intervention helped to build rapport and trust that could help a patient to change their behavior [29]. Although other research has found that self-management may be facilitated by clinic visits [30], in this study, interventionists and patients preferred home visits because they showed that the interventionist was invested in their care, put them at ease, and allowed them to focus on adherence.

The intervention worked through making adherence visible to patients, thereby empowering them to change behaviors. In terms of visibility to others, our study supported research that health care practitioners can be more concerned about negative aspects of surveillance than patients [25], although there was some evidence that those wishing to keep adherence private opted out or withdrew from the pilot RCT. Mohr et al [31] coined the term *supportive accountability* to describe how a trustworthy individual with expertise can support engagement with digital interventions [32]. In our study, this supportive accountability went beyond facilitating engagement and appeared to facilitate outcomes directly. Patients in our study appreciated praise from clinicians when they improved their adherence. Praise is a part of rewards that can facilitate adherence [19,30] by building motivation and self-efficacy [33] and by providing a supportive environment for behavior change [34]. Research has suggested that rewards may work best in combination with identifying barriers and problem solving [35], which both occurred in this intervention.

Vassilev et al [25] suggested that a fit to everyday needs, skills, and daily life was important for intervention success. Research suggests that the fit may help to increase autonomy and thus motivation to change behavior [32]. Our finding that the intervention sometimes did not fit into people’s busy lives is supported by research exploring barriers to adherence in CF where people often cite being too busy to adhere to treatments [19,36-45] and social and work demands creating competing priorities [19,36,46]. This highlights the importance of offering the intervention on an app to enable patients to access adherence data away from their desktop. Some patients also perceived that the educational aspects of the intervention fit better with the needs of younger or newly diagnosed patients who had less knowledge of nebulizer treatments. Research with adolescents and young adults has suggested that they may face similar barriers to adherence as adults [36,38-40,42]. Although the educational aspects of the intervention, such as the videos of how treatments work, may have the potential for use with that group or for those who receive a later diagnosis, we would argue that education alone is unlikely to be sufficient to increase adherence.

We also found perceptions that the intervention fits some groups better than others based on their adherence level with a group of moderate adherers seeming to benefit and engage more than low or high adherers. This could support research into grouping patients by adherence level [11,27], which may have implications for health outcomes [11].

### Strengths and Limitations

This study had some limitations. The sample of patient participants at one of the sites was small because fewer patients agreed to be approached for interview when they were recruited for the pilot RCT. Despite this, we have provided an understanding of patients’ experiences of a new intervention and how the mechanisms of action operated in practice.

### Implications

We modified the intervention based on the results of the wider process evaluation, including the insights raised here [12]. Key implications for the intervention were that the interventionist was a very important aspect of the intervention; there needed to be a feedback mechanism to reward patients for meeting their targets in order to maintain motivation, and that the intervention needed to fit into busy lives, highlighting the need for an app that was produced for use in a future full-scale RCT.

### Conclusions

This behavior change intervention with telehealth components had the expected behavior change mechanisms of action and mechanisms of action associated with effective telehealth interventions for self-management support. The intervention was modified to strengthen mechanisms of action based on these findings, for example, delivery through an app accessed via mobile phones, which is ready for testing in an RCT in 19 UK CF centers.

## Abbreviations

CF: cystic fibrosis
MDT: multidisciplinary team
RCT: randomized controlled trial

## References

[1] Sieben A, van Onzenoort HA, van Laarhoven KJ, Bredie SJ (2016). A multifaceted nurse- and web-based intervention for improving adherence to treatment in patients with cardiovascular disease: rationale and design of the mirror trial. JMIR Res Protoc.

[2] Qian W, Lam TT, Lam HH, Li C, Cheung YT (2019). Telehealth interventions for improving self-management in patients with hemophilia: scoping review of clinical studies. J Med Internet Res.

[3] Tabi K, Randhawa AS, Choi F, Mithani Z, Albers F, Schnieder M, Nikoo M, Vigo D, Jang K, Demlova R, Krausz M (2019). Mobile apps for medication management: review and analysis. JMIR Mhealth Uhealth.

[4] (2019). Cystic Fibrosis FAQs. Cystic Fibrosis Trust - Fighting for a Life Unlimited.

[5] Sawicki GS, Sellers DE, Robinson WM (2009). High treatment burden in adults with cystic fibrosis: challenges to disease self-management. J Cyst Fibros.

[6] Sawicki GS, Tiddens H (2012). Managing treatment complexity in cystic fibrosis: challenges and opportunities. Pediatr Pulmonol.

[7] Daniels T, Goodacre L, Sutton C, Pollard K, Conway S, Peckham D (2011). Accurate assessment of adherence: self-report and clinician report vs electronic monitoring of nebulizers. Chest.

[8] Smith S, Rowbotham N, Regan K (2018). Inhaled anti-pseudomonal antibiotics for long-term therapy in cystic fibrosis. Cochrane Database Syst Rev.

[9] Yang C, Montgomery M (2018). Dornase alfa for cystic fibrosis. Cochrane Database Syst Rev.

[10] Briesacher BA, Quittner AL, Saiman L, Sacco P, Fouayzi H, Quittell LM (2011). Adherence with tobramycin inhaled solution and health care utilization. BMC Pulm Med.

[11] Eakin MN, Bilderback A, Boyle MP, Mogayzel PJ, Riekert KA (2011). Longitudinal association between medication adherence and lung health in people with cystic fibrosis. J Cyst Fibros.

[12] Hind D, Drabble SJ, Arden MA, Mandefield L, Waterhouse S, Maguire C, Cantrill H, Robinson L, Beever D, Scott AJ, Keating S, Hutchings M, Bradley J, Nightingale J, Allenby MI, Dewar J, Whelan P, Ainsworth J, Walters SJ, O'Cathain A, Wildman MJ (2019). Supporting medication adherence for adults with cystic fibrosis: a randomised feasibility study. BMC Pulm Med.

[13] O'Cathain A, Hoddinott P, Lewin S, Thomas KJ, Young B, Adamson J, Jansen YJ, Mills N, Moore G, Donovan JL (2015). Maximising the impact of qualitative research in feasibility studies for randomised controlled trials: guidance for researchers. Pilot Feasibility Stud.

[14] O'Cathain A, Thomas K, Drabble S, Rudolph A, Goode J, Hewison J (2014). Maximising the value of combining qualitative research and randomised controlled trials in health research: the qualitative research in trials (QUART) study--a mixed methods study. Health Technol Assess.

[15] O'Cathain A, Thomas KJ, Drabble SJ, Rudolph A, Hewison J (2013). What can qualitative research do for randomised controlled trials? A systematic mapping review. BMJ Open.

[16] Craig P, Dieppe P, Macintyre S, Michie S, Nazareth I, Petticrew M (2008). Developing and Evaluating Complex Interventions. Medical Research Council - UK Research and Innovation.

[17] Moore G, Audrey S, Barker M, Bond L, Bonell C, Hardeman W, Moore L, O'Cathain A, Tinati T, Wight D, Baird J (2015). Process evaluation of complex interventions: medical research council guidance. Br Med J.

[18] Moore G, Audrey S, Barker M, Bond L, Bonell C, Cooper C, Hardeman W, Moore L, O'Cathain A, Tinati T, Wight D, Baird J (2014). Process evaluation in complex public health intervention studies: the need for guidance. J Epidemiol Community Health.

[19] Arden MA, Drabble S, O'Cathain A, Hutchings M, Wildman M (2019). Adherence to medication in adults with cystic fibrosis: an investigation using objective adherence data and the theoretical domains framework. Br J Health Psychol.

[20] Drabble SJ, O'Cathain A, Arden MA, Hutchings M, Beever D, Wildman M (2019). When is forgetting not forgetting? A discursive analysis of differences in forgetting talk between adults with cystic fibrosis with different levels of adherence to nebulizer treatments. Qual Health Res.

[21] Cane J, O'Connor D, Michie S (2012). Validation of the theoretical domains framework for use in behaviour change and implementation research. Implement Sci.

[22] Michie S, van Stralen MM, West R (2011). The behaviour change wheel: a new method for characterising and designing behaviour change interventions. Implement Sci.

[23] Yardley L, Morrison L, Bradbury K, Muller I (2015). The person-based approach to intervention development: application to digital health-related behavior change interventions. J Med Internet Res.

[24] Wong MD, Sarkisian CA, Davis C, Kinsler J, Cunningham WE (2007). The association between life chaos, health care use, and health status among HIV-infected persons. J Gen Intern Med.

[25] Vassilev I, Rowsell A, Pope C, Kennedy A, O'Cathain A, Salisbury C, Rogers A (2015). Assessing the implementability of telehealth interventions for self-management support: a realist review. Implement Sci.

[26] (2015). English Indices of Deprivation 2019. Government of UK.

[27] Hoo Z, Campbell M, Curley R, Wildman M (2017). An empirical method to cluster objective nebulizer adherence data among adults with cystic fibrosis. Patient Prefer Adherence.

[28] Ritchie J, Spencer L, Bryman A, Burgess B (1994). Qualitative data analysis for applied policy research. Analysing Qualitative Data.

[29] O'Cathain A, Drabble SJ, Foster A, Horspool K, Edwards L, Thomas C, Salisbury C (2016). Being human: a qualitative interview study exploring why a telehealth intervention for management of chronic conditions had a modest effect. J Med Internet Res.

[30] George M, Rand-Giovannetti D, Eakin MN, Borrelli B, Zettler M, Riekert KA (2010). Perceptions of barriers and facilitators: self-management decisions by older adolescents and adults with CF. J Cyst Fibros.

[31] Mohr DC, Cuijpers P, Lehman K (2011). Supportive accountability: a model for providing human support to enhance adherence to eHealth interventions. J Med Internet Res.

[32] Yardley L, Spring BJ, Riper H, Morrison LG, Crane DH, Curtis K, Merchant GC, Naughton F, Blandford A (2016). Understanding and promoting effective engagement with digital behavior change interventions. Am J Prev Med.

[33] Gambling T, Long AF (2010). The realisation of patient-centred care during a 3-year proactive telephone counselling self-care intervention for diabetes. Patient Educ Couns.

[34] Markland D, Ryan R, Tobin V, Rollnick S (2005). Motivational interviewing and self–determination theory. J Soc Clin Psychol.

[35] van Genugten L, Dusseldorp E, Webb TL, van Empelen P (2016). Which combinations of techniques and modes of delivery in internet-based interventions effectively change health behavior? A meta-analysis. J Med Internet Res.

[36] Sawicki GS, Heller KS, Demars N, Robinson WM (2015). Motivating adherence among adolescents with cystic fibrosis: youth and parent perspectives. Pediatr Pulmonol.

[37] Hoo ZH, Boote J, Wildman MJ, Campbell MJ, Gardner B (2017). Determinants of objective adherence to nebulised medications among adults with cystic fibrosis: an exploratory mixed methods study comparing low and high adherers. Heal Psychol Behav Med.

[38] Dziuban EJ, Saab-Abazeed L, Chaudhry SR, Streetman DS, Nasr SZ (2010). Identifying barriers to treatment adherence and related attitudinal patterns in adolescents with cystic fibrosis. Pediatr Pulmonol.

[39] Modi A, Quittner A (2006). Barriers to treatment adherence for children with cystic fibrosis and asthma: what gets in the way?. J Pediatr Psychol.

[40] John EK (2016). Overcoming barriers to treatment adherence in adolescents with cystic fibrosis: a systematic review. Pediatr Neonatal Care.

[41] Llorente RP, García CB, Martín JJ (2008). Treatment compliance in children and adults with cystic fibrosis. J Cyst Fibros.

[42] Bregnballe V, Schiøtz PO, Boisen KA, Pressler T, Thastum M (2011). Barriers to adherence in adolescents and young adults with cystic fibrosis: a questionnaire study in young patients and their parents. Patient Prefer Adherence.

[43] Hogan A, Bonney M, Brien J, Karamy R, Aslani P (2015). Factors affecting nebulised medicine adherence in adult patients with cystic fibrosis: a qualitative study. Int J Clin Pharm.

[44] Lask B (1994). Non-adherence to treatment in cystic fibrosis. J R Soc Med.

[45] Lomas P (2014). Enhancing adherence to inhaled therapies in cystic fibrosis. Ther Adv Respir Dis.

[46] Oddleifson DA, Sawicki GS (2017). Adherence and recursive perception among young adults with cystic fibrosis. Anthropol Med.

